# Editorial: Progress of Translational Medicine in Alzheimer's Disease

**DOI:** 10.3389/fnagi.2022.970044

**Published:** 2022-07-08

**Authors:** Can Zhang, Evandro Fei Fang, Changning Wang

**Affiliations:** ^1^Genetics and Aging Research Unit, Department of Neurology, McCance Center for Brain Health, MassGeneral Institute for Neurodegenerative Disease, Massachusetts General Hospital, Harvard Medical School, Charlestown, MA, United States; ^2^Department of Clinical Molecular Biology, University of Oslo and Akershus University Hospital, Lørenskog, Norway; ^3^The Norwegian Centre on Healthy Ageing (NO-Age), Oslo, Norway; ^4^Department of Radiology, Athinoula A. Martinos Center for Biomedical Imaging, Massachusetts General Hospital, Harvard Medical School, Charlestown, MA, United States

**Keywords:** Alzheimer's disease, translational medicine, biomarker, molecular mechanism, therapeutics, neurodegeneration, neuropathology

## Introduction

More than 50 million individuals worldwide suffer from dementia with ~60–70% affected by Alzheimer's disease (AD). As a progressive and neurodegenerative disorder and the leading cause of dementia in the elderly, AD lacks effective treatments to modify or stop disease progression. Over the past years, studies have identified new molecular mechanisms and modifiable risk factors of AD. Accumulating data suggest that advancing the understanding of AD mechanisms and risk factors may allow the development of targeted interventions to potentially prevent or cure the disease. There are an increasing number of preclinical studies focusing on mechanism-driven therapies, which need to be further characterized before introducing into clinical trials to determine their efficacy.

Here, the Research Topic “*Progress of Translational Medicine in Alzheimer's Disease*” in *Frontiers in Aging Neuroscience* includes a series of 13 articles that discuss recent advances in the field of translational studies in AD and highlight current challenges and outstanding questions requiring further study in order to advance research in this domain. These articles are discussed based on two main specific themes, noted below.

## Theme 1: Studies That Clarify Molecular Etiology of AD

Emerging evidence has shown that exosome-derived biomarkers are potentially related to early occurrence and development of AD, which requires a consolidated analysis. Xing et al. have completed a meta-analysis, combing through discoveries which describe the diagnostic values of exosome-derived biomarkers in AD and MCI (Mild Cognitive Impairment). The meta-analysis includes 19 eligible studies involving 3,742 subjects and supports the potential diagnostic value of exosome-derived biomarkers in AD and MCI, and lays a foundation for future research to further confirm this finding.

Over the past decade, numerous genome-wide association studies (GWAS) have identified significant genes associated with increased risk of AD, for which a systemic review to identify potential molecular mechanisms was overdue. In a review article, Vogrinc et al. summarize more than 100 AD risk loci, with many of them potentially serving as biomarkers of AD progression, even in the preclinical stage of the disease. Furthermore, the analysis of GWAS data has led to identification of key pathways underlying AD pathogenesis, including cellular and metabolic processes, biological regulation, localization, transport, regulation of cellular activities, and neurological system processes. Thus, gene clustering into molecular pathways may help identify novel molecular targets and support the development of more tailored and personalized intervention of AD.

Next, Homann et al. conduct an analysis of GWAS on AD brain imaging biomarkers and neuropsychological phenotypes using the European Medical Information Framework for Alzheimer's Disease Multimodal Biomarker Discovery Dataset (EMIF-AD MBD). Their results highlight the power of using quantitative end phenotypes as outcome traits in AD-related GWAS analyses and nominates new loci underlying cognitive decline.

Although plasma biomarkers for the diagnosis and stratification of AD have been intensively analyzed, no plasma markers have so far been well-established and confirmed for AD diagnosis. To this end, Shi et al. investigate plasma proteomic biomarkers associated with AD through a meta-analysis. The study assesses the diagnostic performance of their own group's previously identified blood biomarkers, and identifies proteins significantly associated with AD, including alpha-2-macroglobulin (A2M), ficolin-2 (FCN2) and fibrinogen gamma chain (FGG).

Presently, another challenging barrier to understanding AD and related dementias is availbility of suitable artificial intelligence (AI) methodologies for analyzing massive AD datasets. In a comprehensive review, Logan et al. explore convolutional neural networks (CNN), a class of deep learning algorithms, for classifying multi-modal neuroimaging data such as magnetic resonance imaging (MRI) and positron emission tomography (PET). These networks may successfully enable early detection of AD and provide insight into AD classification using 3D architectures for multi-modal PET/MRI data.

Recently, increasing evidence has suggested the involvement of gut microbiota dysbiosis in association with AD. In a through review, Wu et al. summarize recent findings involving altered gut microbiota of patients with AD, and discuss pathogenetic mechanisms of gut microbiota in AD, suggesting gut microbiota–targeted therapies for AD.

Despite the long-standing and established findings supporting insoluble protein deposition as pathological hallmarks of AD and other neurodegenerative disorders, studying the reverse process of aggregate disassembly and degradation has only recently gained momentum, following reports of enzymes with distinct aggregate-disassembly activities. A timely review by Mee Hayes et al. discusses recent progress and potential mechanisms for targeting aggregates with proteasomes and disaggregases in liquid droplets.

Furthermore, Stonebarger et al. present an article challenging a major obstacle in understanding the etiology of normative and pathological aged brains in AD—the availability of suitable animal models. This article highlights our current knowledge in the area and examines the use of the rhesus macaque monkey as a pragmatic translational animal model to progress future research in this area.

The issues surrounding the large number of poorly understood underlying functional mechanisms is a major stumbling block in AD research today. Morgan et al. have performed text-mining and generated an exhaustive, systematic assessment of the breadth and diversity of biological pathways within a corpus of 206,324 dementia publication abstracts. The results of this research may be applicable to the context of the broader AD literature corpus.

Finally, Blevins et al. present a review article looking into the AD-related NLRP3 inflammasome, a multiprotein complex that plays a common and pivotal role in regulating innate immunity and inflammation underlying human pathophysiology.

## Theme 2: Studies That Address Potential Therapies For AD

The first article that addresses potential therapies for AD is a study by Zhong et al. focusing on D-penicillamine (D-Pen), a water-soluble metal chelator that can reduce Aβ aggregation. The authors report that D-Pen significantly improves the cognitive functions of APP/PS1 mice and reduces Aβ generation with an associated increase in ADAM10 *via* the non-amyloidogenic processing pathway.

Next, Ma et al. present the results of their study describing the effects and molecular mechanisms of cornel iridoid glycoside (CIG), an active ingredient of the traditional Chinese herb *Cornus officinalis*, in pathological tau P301S transgenic mice. The data support CIG as a promising molecule for potentially treating AD-related tauopathy.

Because type 2 diabetes (T2D) is a major contributor to the development of AD and AD patients with co-occurrence of T2D are regularly observed in the clinical arena, Carranza-Naval et al. analyze liraglutide (LRGT), a glucagon-like peptide-1 agonist, for its effects on reducing pathological changes in the brain of a mixed murine model of AD and T2D (APP/PS1 x db/db mice). These results support the potential for LRGT therapy to reduce AD-associated brain complications.

In closing, we would like to thank all the contributing authors and reviewers for their valuable expertise and effort toward successfully compiling this series of research focusing on translational medicine for AD. We realize that although these selected articles cover a good number of studies reporting up to date research findings, future studies will be necessary to better understand the “elephant in the room:” the etiology of AD, which is our primary goal in developing this Research Topic. We sincerely hope that this Research Topic of articles will provide a useful point of reference and stimulate future studies committed to ultimately understanding the complex pathogenesis of AD and advancing the development of useful therapeutics for AD.

## Author Contributions

All authors listed have made a substantial, direct, and intellectual contribution to the work and approved it for publication.

## Conflict of Interest

EF has CRADA arrangement with ChromaDex and is consultant to Aladdin Healthcare Technologies, Vancouver Dementia Prevention Center, and Intellectual Labs. The remaining authors declare that the research was conducted in the absence of any commercial or financial relationships that could be construed as a potential conflict of interest.

## Publisher's Note

All claims expressed in this article are solely those of the authors and do not necessarily represent those of their affiliated organizations, or those of the publisher, the editors and the reviewers. Any product that may be evaluated in this article, or claim that may be made by its manufacturer, is not guaranteed or endorsed by the publisher.

